# Clinical and genetic analysis in Chinese families with synpolydactyly, and cellular localization of HOXD13 with different length of polyalanine tract

**DOI:** 10.3389/fgene.2023.1105046

**Published:** 2023-03-22

**Authors:** Xiumin Chen, Feiyue Zhao, Yiming Xu, Yixuan Cao, Shan Li, Xue Zhang, Xiuli Zhao

**Affiliations:** Department of Medical Genetics, Institute of Basic Medical Sciences, Chinese Academy of Medical Sciences and School of Basic Medicine, Peking Union Medical College, Beijing, China

**Keywords:** *HOXD13*, synpolydactyly, preaxial polydactyly, polyalanine expansion, variant

## Abstract

Synpolydactyly (SPD) is caused by mutations in the transcription factor gene *HOXD13*. Such mutations include polyalanine expansion (PAE), but further study is required for the phenotypic spectrum characteristics of *HOXD13* PAE. We investigated four unrelated Chinese families with significant limb malformations. Three PAEs were found in the *HOXD13* polyalanine coding region: c.172_192dup (p.Ala58_Ala64dup) in Family 1, c.169_192dup (p.Ala57_Ala64dup) in Family 2, and c.183_210dup (p.Ala62_Ala70dup) in Family 3 and Family 4. Interestingly, we identified a new manifestation of preaxial polydactyly in both hands in a pediatric patient with an expansion of seven alanines, a phenotype not previously noted in SPD patients. Comparing with the wild-type cells and mutant cells with polyalanine contractions (PACs), the HOXD13 protein with a PAE of nine-alanine or more was difficult to enter the nucleus, and easy to form inclusion bodies in the cytoplasm, and with the increase of PAE, the more inclusion bodies were formed. This study not only expanded the phenotypic spectrum of SPD, but also enriched our understanding of its pathogenic mechanisms.

## Introduction

Synpolydactyly (SPD; MIM 186000), also known as syndactyly type Ⅱ, is a distal limb abnormality characterized by fusion of the third and fourth fingers and the fourth and fifth toes and the presence of redundant digits in the syndactylous web(Goodman, 2002). SPD is currently classified into three types: SPD1, SPD2, and SPD3 (Temtamy and McKusick, 1978; Malik and Grzeschik, 2008). Of these, SPD1 is caused by variants in *HOXD13* on chromosome 2q31. *HOXD13* is a member of the HOX family that encodes transcription factors and plays an important role in limb development (Muragaki et al., 1996).

In the first exon of *HOXD13* gene, there is a coding region of polyalanine chain composed of 15 alanines. Expansions of more than 7 to 14 N-terminal polyalanines are pathogenic (Akarsu et al., 1996). Studies of genotypic and phenotypic correlations suggested that the longer the polyalanine tract was, the more severe the phenotype was observed (Goodman et al., 1997). Variations of c.183_206dup, c.186_212dup (Gong et al., 2011), and c.893G>A (Wang et al., 2012) in *HOXD13* and their co-segregation in affected individuals have been reported by several groups (Sun et al., 2021; Wajid et al., 2009; Zaib et al., 2019). The variant c.925A>T in *HOXD13* causes atypical SPD by impairing the downstream transcription of *EPHA7* (Guo et al., 2021). Another pathogenic variant of SPD involves a heterozygous 11,451-bp microdeletion at chr2:176933872-176945322 (GRCh37), which is located upstream of *HOXD13* (Jia et al., 2021). Mutant protein with additional polyalanine expansion (PAE) is expected to destabilize the normal protein structure, leading to aggregation (Wajid et al., 2009). This prevents the protein from being transported from the cytoplasm to the nucleus, where it normally acts as a transcription factor, although its regulatory pathway and downstream genes remain unknown. In addition, PAE causes cytoplasmic aggregates of HOXD13 molecules and may inhibit the translocation of wild-type (WT) proteins to the nucleus, suggesting that PAE in *HOXD13* may exhibit dominant-negative effects. To date, 52 variants were recorded in the professional edition (Human Gene Mutation Database, HGMD, 2021.4), and 18 variants were recorded responsible for syndactyly. Further studies on the pathogenic mechanism of PAEs in SPD are needed, including the identification of novel phenotypes regulated by *HOXD13* during PAE.

In this study, we recruited four families with SPD and discovered a novel presentation of preaxial polydactyly of both hands in a pediatric patient. We identified the effect of polyalanine changes on cellular localization of HOXD13 proteins, by constructing the full length cDNA vectors of wild type and mutant *HOXD13* with different length of polyalanine. Our findings expand the phenotypic spectrum and advance our understanding of human limb development.

## Materials and methods

### Patients

**FIGURE 1:**
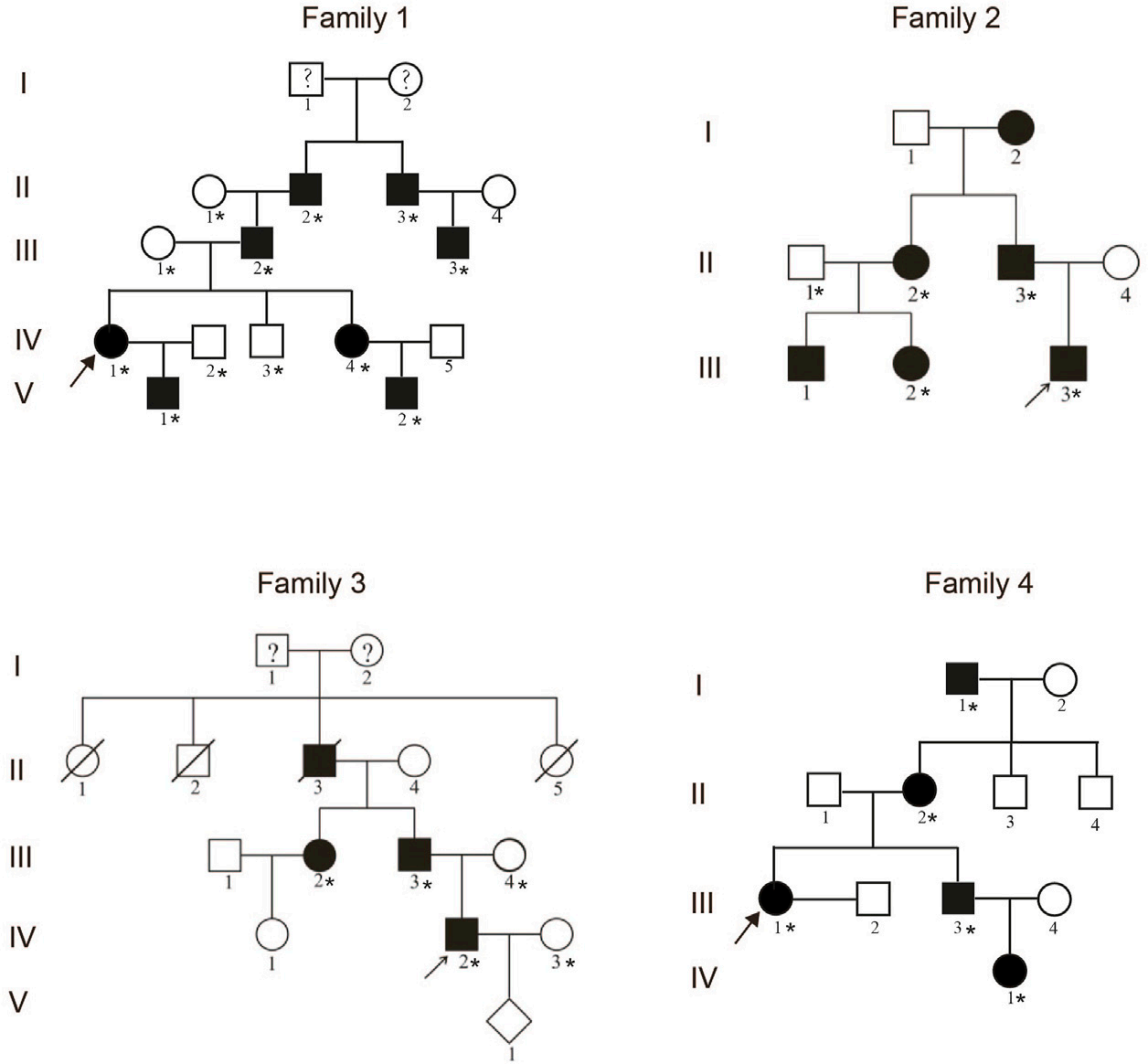
Pedigrees of four unrelated families with the synpolydactyly. The arrows indicate the proband. The asterisks represent the individuals in the families who provided peripheral blood samples.

A total of 16 patients from four unrelated Chinese families living in mainland China were recruited in this study (Figure 1). All of these patients had varying degrees of clinical manifestations of SPD1. After obtaining the approval of the Institutional Review Board of the Chinese Academy of Medical Sciences and School of Basic Medicine (032-2017) and the informed consent of all participants, we collected peripheral blood samples from these patients and their available family members.

### Mutation analysis

Genomic DNA of the family members was extracted from peripheral blood by the standard SDS proteinase K/phenol/chloroform method. The coding exons and exon–intron boundaries of *HOXD13* (NM_000523.3) were amplified by polymerase chain reaction (PCR). Primer sequences were as follows: forward, 5′-CTTTCTCTCCGCGCCTGTGTTC-3′, and reverse, 5′-CTACAACGGCAGAAGAGGACGACG-3′. The PCR volume comprised 50–100 ng genomic DNA, 0.5 μL of each primer (10 μM), 4 μL dNTP (2.5 mM), 12.5 μL of 2 × GC buffer Ⅱ, 2.5 U LA Taq DNA polymerase (Takara Biotechnology Co., Ltd., Dalian, China), and deionized water to 25 μL. The PCR was performed according to the following conditions: 95°C for 3 min; 35 cycles at 94°C for 30 s, 65°C for 30 s, and 72°C for 30 s; and a final extension for 8 min at 72°C. Sanger DNA sequencing was performed by a commercial service. The reference sequence of the candidate gene *HOXD13* (NM_000523.4) was obtained from the UCSC Genome Browser (http://genome.ucsc.edu). The sequencing data were analyzed using CodonCode Aligner software (version 6.0.2.6; CodonCode Corporation, Centerville, MA, United States).

T clone sequencing was performed to detect the sequence of the GCN trinucleotide repeat encoding the polyalanine tract. Briefly, the PCR product was ligated into pMD19-T vector; the ligation reaction volume included 4 μL Solution Ⅰ, 1 μL pMD19-T vector (Takara Biotechnology Co., Ltd.; Code: D102A), 1 μL PCR product (50 ng), and 4 μL sterile deionized water; the ligation reaction was performed at 16 °C for 15 min. The ligation product was then transformed into *E. coli* DH5α, with 42°C heat shock for 90 s and plate culture for 16 h pMD19-HOXD13-PolyA plasmid DNA was extracted using a Qiagen Plasmid Mini kit (12125) and then cloning-sequencing was performed using DNA Sanger sequencing.

### Mutation validation

The PAE was verified by PCR combined with 8% neutral polyacrylamide gel electrophoresis and conventional silver staining (0.1% AgNO3) in all available members of Family 1. And PAEs in other three families with SPD were confirmed using PCR and 3% agarose gel electrophoresis.

### *HOXD13* protein localization

Full length human *HOXD13* cDNA of the wild type and mutant types with polyalanine contraction (PAC: −13A, −11A, −7A) and polyalanine expansion (PAE: +9A, +14A, +17A) were cloned into the eukaryotic expression vector pEGFP-C3. The mutant plasmids were constructed by insertion of synthesized oligonucleotides including the different length of polyalanine tract and confirmed by Sanger sequencing. COS-7 cells were cultured in DMEM (Invitrogen) supplemented with 1% penicillin/streptomycin and 10% fetal bovine serum. At a cell confluence of 75%, the wild type and six mutant plasmids were transfected into COS-7 cells, respectively. The transfection was performed in a 24-well plate using Lipofectamine 2000 (Invitrogen) according to the manufacturer’s instructions. The transfected cells were observed 24 h after transfection and imaged using an Olympus IX71 microscope (Zhao, 2006). The localization and intensity of GFP fluorescence signal in COS-7 cells were evaluated and analyzed.

## Results

### Clinical report

**FIGURE 2:**
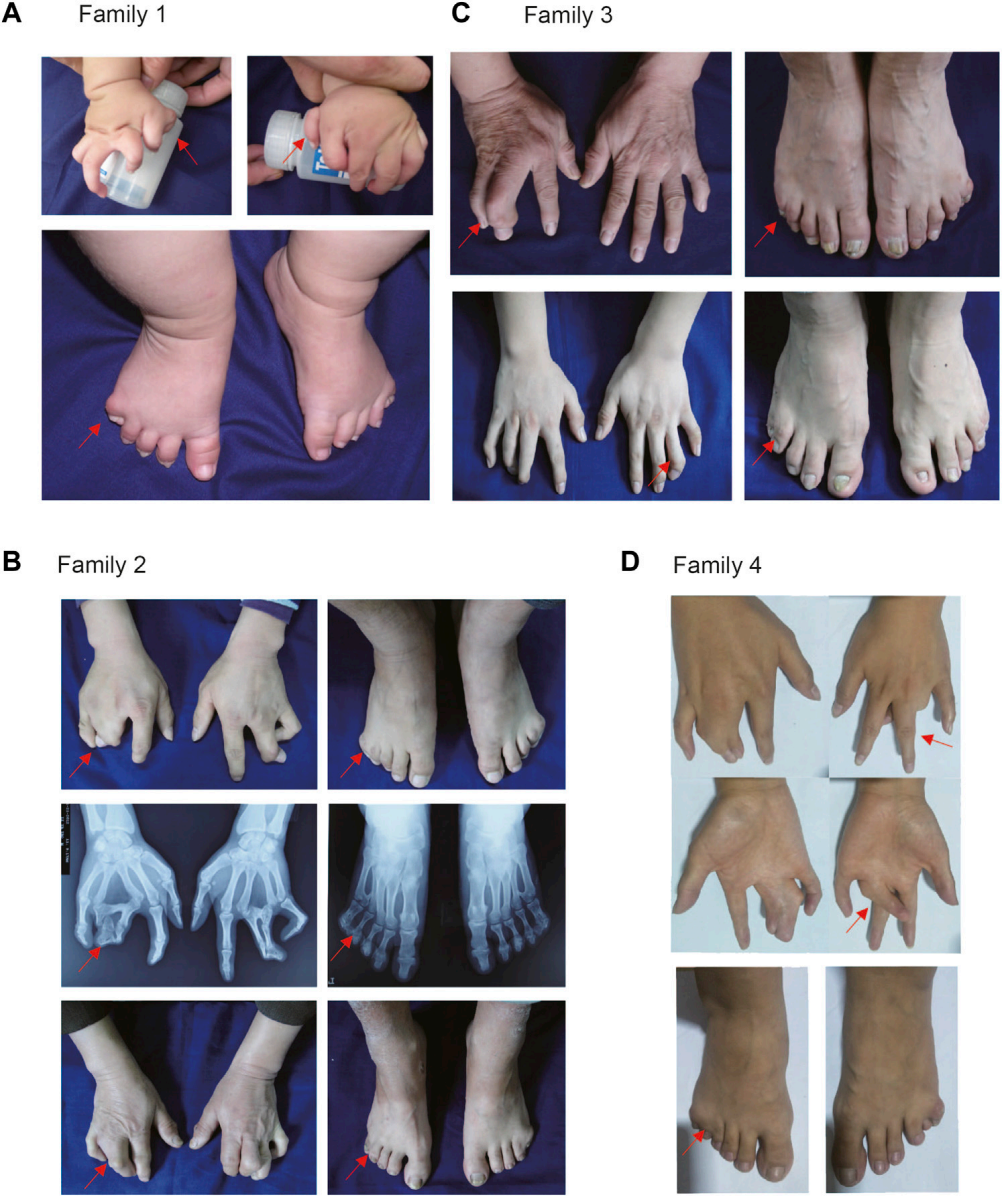
Clinical manifestation of patients with synpolydactyly. **(A)** Bilateral preaxial polydactyly of hands and postaxial synpolydactyly of feet found in a pediatric patient. **(B)** Bilateral or unilateral synpolydactyly in 3/4 fingers of the affected individuals in SPD families. **(C)** Congenital syndactyly between the 3/4 fingers. **(D)** There were syndactyly in the proband’s 3/4 fingers. The arrows indicate the patients’ fingers.

In Family 1, all male patients had severe hand and foot deformity, whereas female patients had only mild membranous syndactyly. Interestingly, preaxial polydactyly in both hands was found in a young patient, which has never been reported in patients with PAE of *HOXD13* (Figure 2A). In Family 2, fingers 3/4 of the proband were syndactyly, with bilateral symmetry. The proximal fingers were fused, forming a severe 3/4 finger deformity, with slight differences on both sides. The patients had bilateral bifurcation of the fourth metatarsal, bony polydactyly of the fifth fingers, and periosteum polydactyly of fingers 4/5 (Figure 2B). In Family 3, the proband was born with a congenital 3/4-finger syndactyly deformity of her right hand and underwent orthopedic surgery at age 3 (Figure 2C). In Family 4, the proband was completely synpolydactyly with fingers 3/4 and toes 4/5 (Figure 2D). The synpolydactyly in toes 4/5 were similar in all families (Figures 2A–D).

### Mutation identification

**FIGURE 3:**
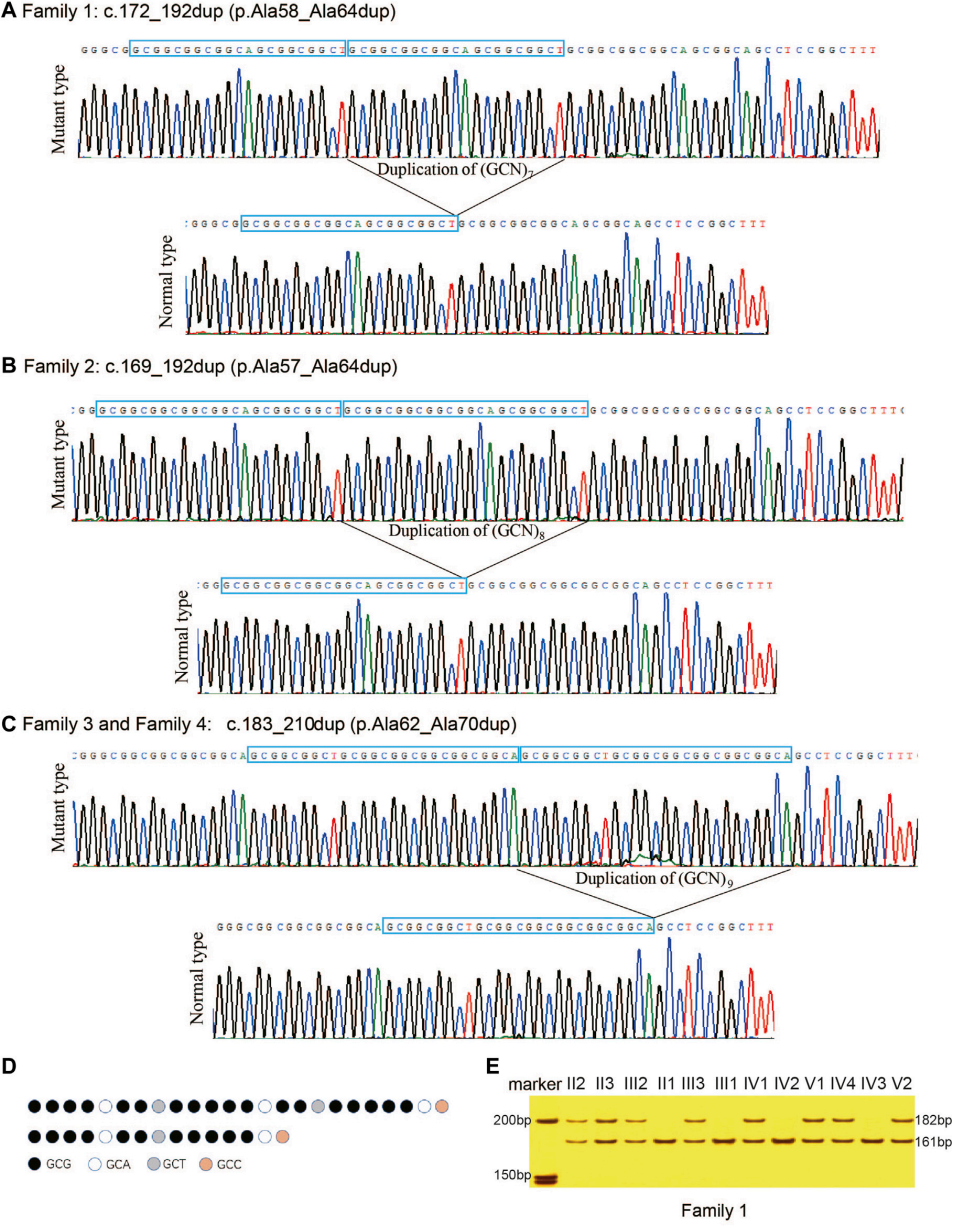
Polyalanine expansions of *HOXD13* identified in the families with synpolydactyly. **(A)** Expansion of seven-alanine, c.172_192dup (p.Ala58_Ala64dup), in Family 1. **(B)** Expansion of eight-alanine, c.169_192dup (p.Ala57_Ala64dup), in Family 2. **(C)** Expansion of nine-alanine, c.183_210dup (p.Ala62_Ala70dup), in Family 3 and Family 4. **(D)** Take Family 4 as an example, pattern diagram of different codons in *HOXD13* polyalanine tract of the normal allele and mutant allele with expansion of nine-alanine. **(E)** Two bands (182bp and 161bp) were separated on 8% neutral polyacrylamide gel. The result showed that all patients had the duplication.

The PAEs found in *HOXD13* of the probands were confirmed using PCR combined with cloning-sequencing. Three different duplications of triplet repeat were identified in the N-terminal polyalanine coding region in *HOXD13*: c.172_192dup (p.Ala58_Ala64dup) in Family 1 (Figure 3A), c.169_192dup (p.Ala57_Ala64dup) in Family 2 (Figure 3B), and c.183_210dup (p.Ala62_Ala70dup) in Family 3 and Family 4 (Figure 3C). The pattern diagram of different codons in *HOXD13* polyalanine tract of the normal allele and mutant allele with expansion of nine-alanine was shown in (Figure 3D).

### Mutation validation

In the four families of the current study, all the affected individuals with SPD were heterozygotes of PAE in *HOXD13*, and all the unaffected individuals harbored only one allele with a length of 161bp. All the patients with SPD had two alleles, including one 161bp fragment and a longer fragment with PAE. The results of polyacrylamide gel electrophoresis showed that all the patients in Family 1 had a normal allele of 161bp and a mutant allele of 182bp (Figure 3E), all SPD patients in Family 2 included fragments of 161bp and 185bp, and all the affected in Family 3 and Family 4 harbored two fragments with length of 161bp and 188bp (results of the agarose gel electrophoresis were not provided in this publication). Co-segregation of PAE variants with the SPD phenotype was confirmed in all four families.

### Cellular localization of mutant *HOXD13* protein

**FIGURE 4:**
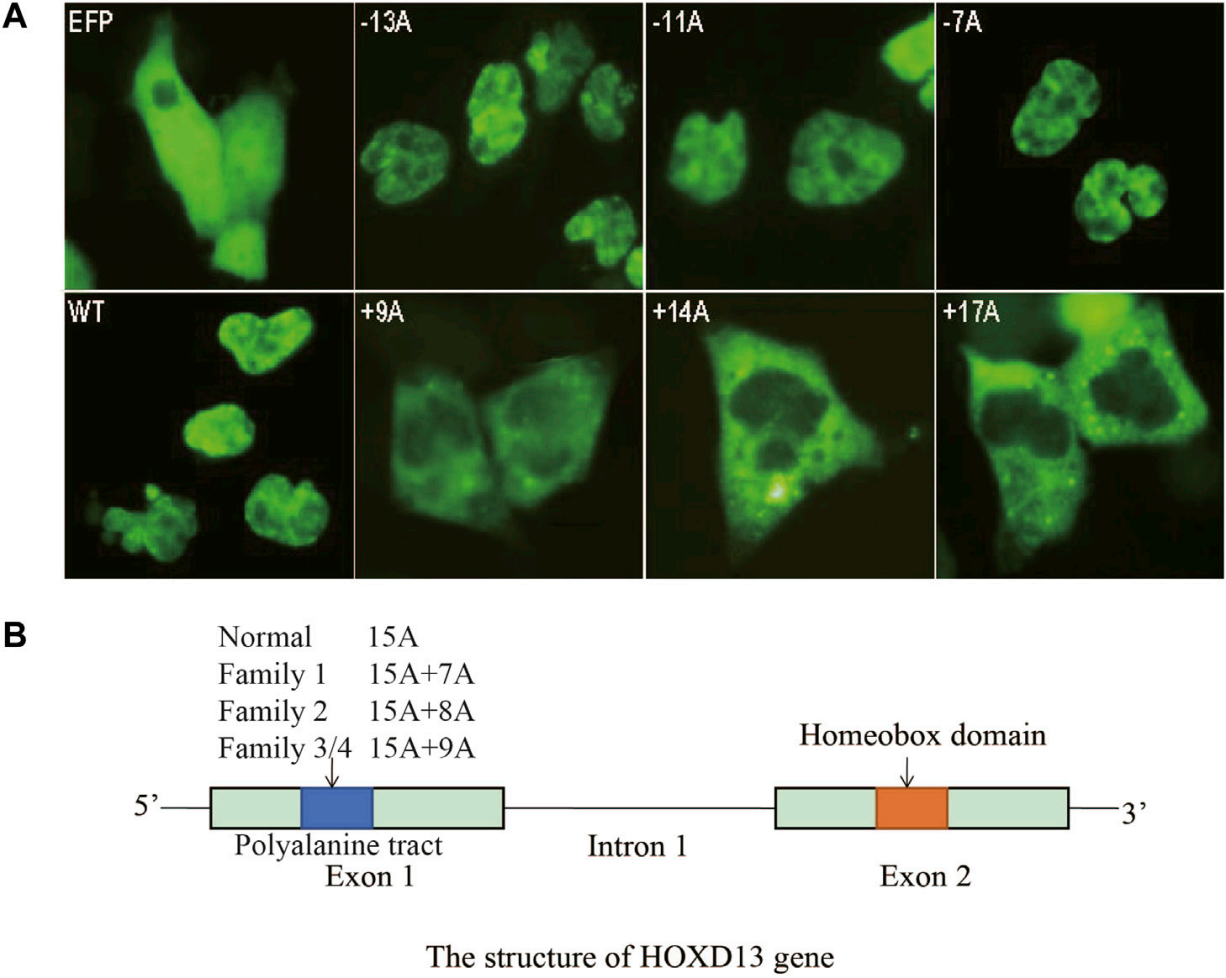
Cellular localization of the fusion protein of GFP-HOXD13 in COS-7 cells. **(A)** Expression of eight vectors: GFP, pEGFP-C3 vector; WT, the pEGFP-C3 with wild type HOXD13; three HOXD13 poly-Ala mutant plasmids with polyalanine contractions (PACs) (−13A, −11A and -7A); three HOXD13 poly-Ala mutant plasmids with polyalanine expansions (PAEs) (+9A, +14A, and +17A). The wild type and the PAC mutant of HOXD13 did not affect the cellular localization of the fusion protein, but the PAE of HOXD13 caused the aggregation of fusion proteins in the cytoplasm. Moreover, with the increase of PAE, the inclusion body of fusion protein in cytoplasm increased. **(B)** Schematic structure of *HOXD13*, the polyalanine tract located in the first exon and the homeobox domain located in the second exon.

As for the effects of polyalanine length on *HOXD13* protein entering the nucleus, COS-7 cells were transfected with plasmids containing *HOXD13* WT, PAC (−13A, −11A, −7A), and PAE (+9A, +14A, +17A). Cells transfected with the polyalanine of +14A and +17A PAE in *HOXD13* showed the strongest GFP signal, whereas the GFP signal appeared in the nucleus for a short period of time and was weak. GFP-HOXD13 fusion protein was mainly distributed in the cytoplasm and nucleus, but most cells had stronger signal in the cytoplasm (Figure 4A). There was no significant relationship between PAC mutations and the entering into the nucleus. The GFP-fusion proteins with PAE were distributed in the nucleus and cytoplasm, and increased with the increase of PAE length (Figure 4A). It is likely due to the accumulation of fusion proteins in the cytoplasm instead of in the nucleus. These results indicated that PAE affected the entering of *HOXD13* protein into the nucleus, and this effect was gradually aggravated with the increase of PAE length. A schematic of *HOXD13* shows two exons with the poly-Ala tract and an intron (Figure 4B).

## Discussion

Homeobox genes play important roles in embryo development and encode a highly conserved family of transcription factors. *HOXD13* is the 5’ terminal member of the HOXD cluster and has two coding exons. The first exon contains an incomplete trinucleotide repeat of GCN (N represents A/C/G/T), which encodes a polyalanine tract of 15-alanine. The second exon encodes a highly conserved homeobox domain including a nuclear localization signal (Akarsu et al., 1996). Point mutations, frameshift mutations, or PAE in the first exon of human *HOXD13* can lead to the typical SPD phenotype, and the missense mutations in the second exon led to brachydactyly type E and syndactyly type V (Zhao et al., 2007). In the present study, three PAEs in *HOXD13* were found in four unrelated Chinese families: c.172_192dup (p.Ala58_Ala64dup) in Family 1, c.169_192dup (p.Ala57_Ala64dup) in Family 2, and c.183_210dup (p.Ala62_Ala70dup) in Family 3 and Family 4. Correlation between the severity of SPD and the PAE length of *HOXD13* has been discussed for many years (Chintalaphani et al., 2021). The expansion of two other alanines does not have pathological consequences (Malik et al., 2007), and to our knowledge, PAE less than 7 alanines has not been reported in human, this means that synpolydactyly can be caused only when polyalanine expansion reaches a certain threshold, such as a PAE of seven alanine or more (Malik and Grzeschik, 2008). Moreover, in our study, it was found that the male patients showed more severe limb malformation than that of female patients in all families. Gong et al. reported a PAE of c. 186-212dup (p.Ala63_Ala71dup), in the exon 1 of *HOXD13*, which resulted in variable expressivity, such as SPD, camptodactyly, symphalangism, transverse phalanx, and osseous fusion of the third metacarpal with the proximal phalanx (Gong et al., 2011). This means that variable expressivity between different genders was an important genetic characteristics of SPD. We suggest that variable expressivity of SPD may be related to hormone level. Why this phenotypic heterogeneity occurs remains to be elucidated, and potential modifier genes are also an issue requiring attention in further research.

HOXD13 is a transcription factor that must enter the nucleus to regulate downstream target genes (Jung et al., 2004). Thus, HOXD13 protein may have a nuclear localization signal (NLS) (Williams et al., 2005). The PAE exceeding seven alanines disrupts this functional domain, leading to aggregation of the protein and forming inclusion bodies in cytoplasm and thereby prevents the HOXD13 entering the nucleus (Albrecht & Mundlos, 2005), thus influences its regulatory role on downstream genes. Moreover, with the increase of PAE length, GFP-HOXD13 protein aggregates more in the cytoplasm and forms more inclusion bodies. On the other hand, the longer the PAE is, the less the fusion protein appears in the nucleus. However, there was no difference in the localizations of GFP-HOXD13 fusion protein between cells with PAC group and wild-type, and the fluorescence signal was mainly located in the nucleus. The present study suggests that PAE affects the nucleation of HOXD13 protein and that the effect intensifies with an increase of PAE length.

In summary, we identified a new manifestation of symmetrical preaxial polydactyly hands in a pediatric patient with a seven-alanine expansion, a phenotype not mentioned previously in patients with *HOXD13* PAE. PAE more than 7 alanines affect the nucleation of *HOXD13* protein and the effect became more notable with the extension of PAE. These findings expand the phenotypic spectrum and advance our understanding of the mechanism of human synpolydactyly.

## Data Availability

The original contributions presented in the study are included in the article/supplementary material, further inquiries can be directed to the corresponding author.

## References

[B1] Akarsu A. N. Stoilov I. Yilmaz E. Sayli B. S. Sarfarazi M. (1996). Genomic structure of HOXD13 gene: A nine polyalanine duplication causes synpolydactyly in two unrelated families. Hum. Mol. Genet. 5 (7), 945–952. 10.1093/hmg/5.7.945 8817328

[B2] Albrecht A. Mundlos S. (2005). The other trinucleotide repeat: Polyalanine expansion disorders. Curr. Opin. Genet. Dev. 15 (3), 285–293. 10.1016/j.gde.2005.04.003 15917204

[B3] Chintalaphani S. R. Pineda S. S. Deveson I. W. Kumar K. R. (2021). An update on the neurological short tandem repeat expansion disorders and the emergence of long-read sequencing diagnostics. Acta Neuropathol. Commun. 9 (1), 98. 10.1186/s40478-021-01201-x 34034831 PMC8145836

[B4] Gong L. Wang B. Wang J. Yu H. Ma X. Yang J. (2011). Polyalanine repeat expansion mutation of the HOXD13 gene in a Chinese family with unusual clinical manifestations of synpolydactyly. Eur. J. Med. Genet. 54 (2), 108–111. 10.1016/j.ejmg.2010.10.007 20974300

[B5] Goodman F. R. (2002). Limb malformations and the human HOX genes. Am. J. Med. Genet. 112 (3), 256–265. 10.1002/ajmg.10776 12357469

[B6] Goodman F. R. Mundlos S. Muragaki Y. Donnai D. GiovannucciUzielli M. L. Lapi E. (1997). Synpolydactyly phenotypes correlate with size of expansions in HOXD13 polyalanine tract. Proc. Natl. Acad. Sci. U. S. A. 94 (14), 7458–7463. 10.1073/pnas.94.14.7458 9207113 PMC23843

[B7] Guo R. J. Fang X. Mao H. L. Sun B. Zhou J. T. An Y. (2021). A novel missense variant of HOXD13 caused atypical synpolydactyly by impairing the downstream gene expression and literature Review for genotype-phenotype correlations. Front. Genet. 12, 731278. 10.3389/fgene.2021.731278 34777468 PMC8579070

[B8] Jia W. M. Zhou X. P. Guo N. A. Zhang D. Z. Hou M. Q. Luo Y. L. (2021). A novel microdeletion upstream of *HOXD13* in a Chinese family with synpolydactyly. Am. J. Med. Genet. Part A 188, 31–36. 10.1002/ajmg.a.62480 34467619

[B9] Jung C. Kim R. S. Zhang H. J. Lee S. J. Jeng M. H. (2004). HOXB13 induces growth suppression of prostate cancer cells as a repressor of hormone-activated androgen receptor signaling. Cancer Res. 64 (24), 9185–9192. 10.1158/0008-5472.CAN-04-1330 15604291

[B10] Malik S. Girisha K. M. Wajid M. Roy A. K. Phadke S. R. Haque S. (2007). Synpolydactyly and HOXD13 polyalanine repeat: Addition of 2 alanine residues is without clinical consequences. Bmc Med. Genet. 8, 78. 10.1186/1471-2350-8-78 18072967 PMC2222244

[B11] Malik S. Grzeschik K. H. (2008). Synpolydactyly: Clinical and molecular advances. Clin. Genet. 73 (2), 113–120. 10.1111/j.1399-0004.2007.00935.x 18177473

[B12] Muragaki Y. Mundlos S. Upton J. Olsen B. R. (1996). Altered growth and branching patterns in synpolydactyly caused by mutations in HOXD13. Science 272 (5261), 548–551. 10.1126/science.272.5261.548 8614804

[B13] Sun L. Y. Huang Y. Z. Zhao S. Zhao J. H. Yan Z. H. Guo Y. (2021). Deciphering the mutational signature of congenital limb malformations. Mol. Therapy-Nucleic Acids 24, 961–970. 10.1016/j.omtn.2021.04.012 PMC8141661 34094714

[B14] Temtamy S. A. McKusick V. A. (1978). The genetics of hand malformations. Birth Defects Orig. Artic. Ser. 14 (3), 1–619. 215242

[B15] Wajid M. Ishii Y. Kurban M. Dua-Awereh M. B. Shimomura Y. Christiano A. M. (2009). Polyalanine repeat expansion mutations in the HOXD13 gene in Pakistani families with synpolydactyly. Clin. Genet. 76 (3), 300–302. 10.1111/j.1399-0004.2009.01213.x 19686284

[B16] Wang B. Xu B. Cheng Z. Zhou X. Wang J. Yang G. (2012). A novel non-synonymous mutation in the homeodomain of HOXD13 causes synpolydactyly in a Chinese family. Clin. Chim. Acta 413 (13-14), 1049–1052. 10.1016/j.cca.2012.02.015 22374128

[B17] Williams T. M. Williams M. E. Heaton J. H. Gelehrter T. D. Innis J. W. (2005). Group 13 HOX proteins interact with the MH2 domain of R-Smads and modulate Smad transcriptional activation functions independent of HOX DNA-binding capability. Nucleic Acids Res. 33 (14), 4475–4484. 10.1093/nar/gki761 16087734 PMC1183491

[B18] Zaib T. Ji W. Saleem K. Nie G. Li C. Cao L. (2019). A heterozygous duplication variant of the HOXD13 gene caused synpolydactyly type 1 with variable expressivity in a Chinese family. BMC Med. Genet. 20 (1), 203. 10.1186/s12881-019-0908-6 31870337 PMC6929446

[B19] Zhao X. (2006). Molecular Genetics of limb malformations: Mutation identification in HOXD13, TNNI2 and TPM2 [D]. Beijing: Peking Union Medical College.

[B20] Zhao X. Sun M. Zhao J. Leyva J. A. Zhu H. Yang W. (2007). Mutations in HOXD13 underlie syndactyly type V and a novel brachydactyly-syndactyly syndrome. Am. J. Hum. Genet. 80 (2), 361–371. 10.1086/511387 17236141 PMC1785357

